# Modulation of Multiple Gene Clusters’ Expression by the PAS-LuxR Transcriptional Regulator PteF

**DOI:** 10.3390/antibiotics11080994

**Published:** 2022-07-24

**Authors:** Cláudia M. Vicente, Tamara D. Payero, Antonio Rodríguez-García, Eva G. Barreales, Antonio de Pedro, Fernando Santos-Beneit, Jesús F. Aparicio

**Affiliations:** 1 Department of Molecular Biology, Area of Microbiology, Faculty of Biology, Universidad de León, 24071 León, Spain; claudia.vicente@insa-toulouse.fr (C.M.V.); tdiep@unileon.es (T.D.P.); arodg@unileon.es (A.R.-G.); egarcb@unileon.es (E.G.B.); apedl@unileon.es (A.d.P.); 2 Institute of Biotechnology INBIOTEC, Parque Científico de León, Avda. Real, No 1, 24006 León, Spain; fernando.santos.beneit@uva.es; 3 Toulouse Biotechnology Institute (TBI), CNRS, INRAE, INSA, Université de Toulouse, 31077 Toulouse, France; 4 Environmental Technology, Institute of Sustainable Processes, University of Valladolid, 47011 Valladolid, Spain

**Keywords:** antifungal agent, gene regulation, LuxR, PAS domain, polyene macrolide, *Streptomyces*

## Abstract

PAS-LuxR transcriptional regulators are conserved proteins governing polyene antifungal biosynthesis. PteF is the regulator of filipin biosynthesis from *Streptomyces avermitilis*. Its mutation drastically abates filipin, but also oligomycin production, a macrolide ATP-synthase inhibitor, and delays sporulation; thus, it has been considered a transcriptional activator. Transcriptomic analyses were performed in *S. avermitilis* Δ*pteF* and its parental strain. Both strains were grown in a YEME medium without sucrose, and the samples were taken at exponential and stationary growth phases. A total of 257 genes showed an altered expression in the mutant, most of them at the exponential growth phase. Surprisingly, despite PteF being considered an activator, most of the genes affected showed overexpression, thereby suggesting a negative modulation. The affected genes were related to various metabolic processes, including genetic information processing; DNA, energy, carbohydrate, and lipid metabolism; morphological differentiation; and transcriptional regulation, among others, but were particularly related to secondary metabolite biosynthesis. Notably, 10 secondary metabolite gene clusters out of the 38 encoded by the genome showed altered expression profiles in the mutant, suggesting a regulatory role for PteF that is wider than expected. The transcriptomic results were validated by quantitative reverse-transcription polymerase chain reaction. These findings provide important clues to understanding the intertwined regulatory machinery that modulates antibiotic biosynthesis in *Streptomyces*.

## 1. Introduction

Polyene macrolide antifungals are natural products produced by *Streptomycetes* and related bacteria. These are filamentous soil-dwellers that undergo a complex life cycle involving differentiation and sporulation and they are well known for their ability to produce an impressive array of bioactive compounds. The control of these compounds’ production is a rather complex process involving multiple levels of intertwined regulation. Typically, the lowest level is governed by pathway-specific transcriptional regulators, which are encoded within the respective biosynthetic gene clusters.

PAS-LuxR regulators are transcription factors that combine an N-terminal PAS sensory domain [1] with a C-terminal helix-turn-helix (HTH) motif of the LuxR type for DNA-binding [2]. The sensory domain is thought to detect a physical or chemical stimulus and regulate, in response, the activity of the effector domain [3]. The archetype of this class of regulators, PimM, was first identified in the antifungal pimaricin biosynthetic gene cluster from *Streptomyces natalensis* [4]. It was characterized as a transcriptional activator of pimaricin biosynthesis because antifungal production was abolished upon gene deletion, and later, its mode of action was characterized at the molecular level [5]. Since its discovery, homologous regulatory proteins have been found to be encoded in all the known biosynthetic gene clusters of antifungal polyketides (polyenes), and they have been shown to be functionally equivalent, to the extent that the production of pimaricin is restored in *S. natalensis* Δ*pimM* upon the introduction of heterologous regulators of the PAS-LuxR class, such as *nysRIV* (nystatin), *amphRIV* (amphotericin), or *pteF* (filipin), into the strain [6]. Furthermore, the introduction of a single copy of *pimM* into the amphotericin-producing strain *S. nodosus*, into the filipin-producing strain *S. avermitilis*, or into the rimocidin producing strain *S. rimosus*, boosted the production of all polyenes, thus indicating that these regulators are fully exchangeable [6]. Interestingly, these regulatory proteins have only been found to be encoded in polyene gene clusters, in which they participate as the final transcriptional regulator of the regulatory cascade leading to antifungal biosynthesis.

Although PAS-LuxR regulators were initially considered pathway-specific transcriptional regulators due to their location in the chromosome, recent results have shown that they should be considered regulators with a wider range of implications. The canonical operator of PimM was used to search for putative targets of the orthologous protein PteF in the genome of *S. avermitilis*, finding multiple binding sites located inside or upstream from genes involved in different aspects of both primary and secondary metabolism [7], thus suggesting that the regulator could govern those processes. These included genetic information processing, DNA replication and repair, energy metabolism, carbohydrate metabolism, lipid metabolism, morphological differentiation, transcriptional regulation, and secondary metabolite biosynthesis, among others. Several of these operators were selected, and their binding to PimM DNA-binding domain was demonstrated by electrophoretic mobility shift assays (EMSAs). As a proof of concept, the biosynthesis of the ATP-synthase inhibitor oligomycin, whose gene cluster included two operators, was studied [7]. The *pteF*-deleted mutants, who showed a severe loss of filipin production and delayed spore formation in comparison to that of the wild-type strain [8], also showed a severe loss of oligomycin production and reduced expression of *olm* genes. Gene complementation of the mutant restored the phenotype; thus, PteF was able to co-regulate the biosynthesis of two related secondary metabolites, the polyketide macrolides filipin and oligomycin [7]. Therefore, this cross-regulation could be extended to all the clusters where operators were found, which suggests that PAS-LuxR regulators may affect a plethora of processes previously unforeseen. In this sense, the introduction of PAS-LuxR regulatory genes into different *Streptomyces* hosts has already proven useful for the awakening of dormant secondary metabolite biosynthetic genes [9,10].

Herein, we have used microarrays to study the transcriptome of an *S. avermitilis* Δ*pteF* mutant in comparison with that of its parental strain in order to deepen our knowledge about the processes in which PteF is involved, corroborating our previous results and providing the first evidence that PAS-LuxR regulators can behave as wide domain regulators and control the expression of multiple genes, either directly or indirectly, not only related to secondary metabolism but also to essential cellular functions. Their implication in the regulation of several secondary metabolite gene clusters is particularly noteworthy.

## 2. Results and Discussion

### 2.1. Identification of Genes with an Altered Expression Profile in S. avermitilis ΔpteF Mutant

**Figure 1:**
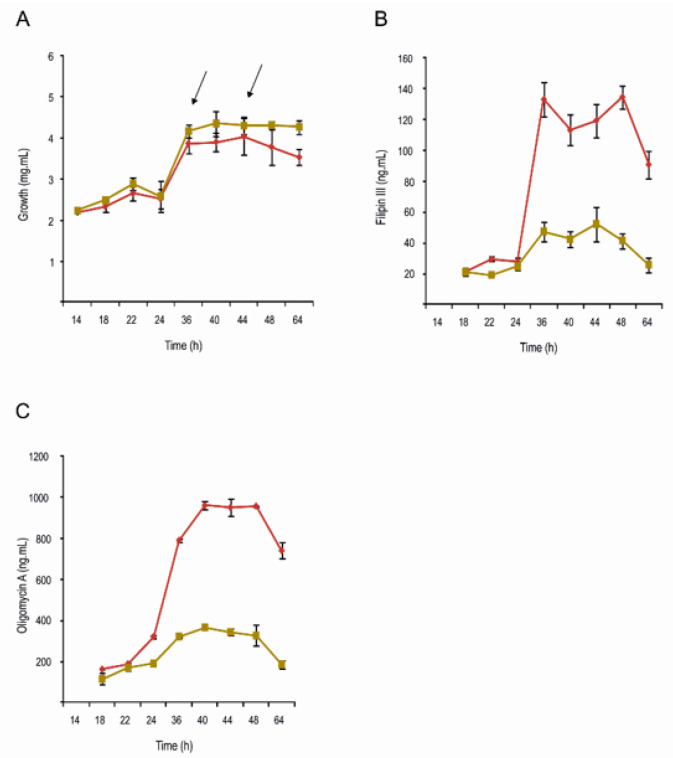
Growth and antibiotic production in YEME medium without sucrose. Strains *S. avermitilis* wt (red), and Δ*pteF* mutant (ochre). (**A**) Growth curves; (**B**) Filipin production; (**C**) Oligomycin production. Arrows indicate RNA samples’ harvesting times.

**Figure 2:**
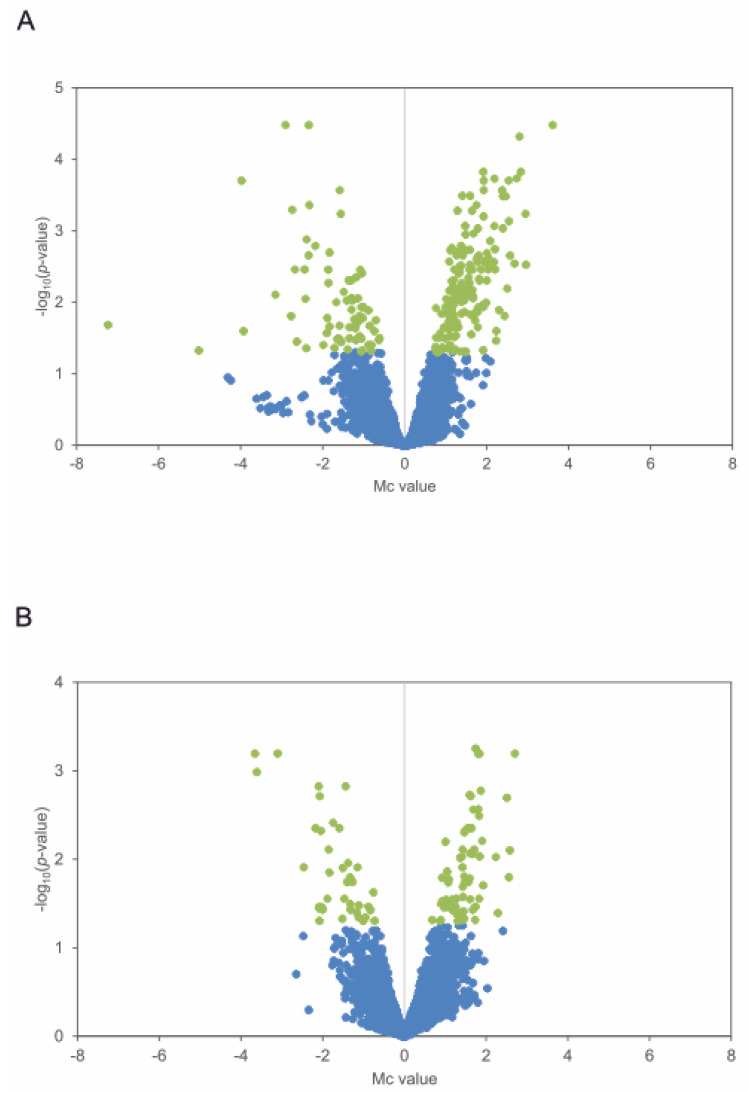
Differentially expressed genes in the mutant strain Δ*pteF*. Volcano plots show differential gene expression distribution during exponential phase (**A**) and stationary phase (**B**). Statistically significant genes are shown in green (log_10_ *p*-value ≥ 1.3).

*S. avermitilis* Δ*pteF* and its parental strain *S. avermitilis* NRRL 8165 were grown in a YEME medium without sucrose, and samples were taken at the end of the exponential and at the middle of the stationary growth phases (Figure 1). A transcriptomic analysis was performed by microarray hybridization to assess the genes with an altered expression in the mutant when compared with the parental strain at two different times during the growth curve. Given that PteF has been demonstrated to control filipin and oligomycin production as well as have an impact on sporulation [7,8], the sampling times were selected to coincide with the onset of secondary metabolite production and with the metabolic changes linked with morphological differentiation, namely, at the end of exponential phase (t1) and early stationary phase of growth (t2). The genomic DNA was used as a universal reference for all the hybridizations. A result was considered statistically significant if the BH-corrected *p*-value was <0.05. It is worth noting that these conditions are quite stringent, given that the genes that constituted direct targets of PteF (e.g., the filipin polyketide synthases *pteA1* and *pteA2*; [8]) were not statistically significant. With this criterion, a microarrays analysis showed significant differences (with a fold change above or below ±2) in the expression of 208 genes of the *pteF*-negative mutant at the end of the exponential phase, and 99 at the stationary phase of growth (Table 1; Figure 2).

Surprisingly, the lack of PteF resulted in the overexpression of a majority of the differentially transcribed genes, at both sampling times, thus indicating that this regulator acts as a negative modulator for the expression of those genes. This was unexpected given that PteF is an activator of both the antifungal filipin [8] and the ATP-synthase inhibitor oligomycin’s [7] biosynthesis.

These genes were related to different cellular processes, including genetic information processing; energy, carbohydrate, and lipid metabolism; DNA replication and repair; morphological differentiation; and transcriptional regulation, among others, but particularly to secondary metabolite biosynthesis (Table 1).

#### 2.1.1. Genes Involved in Genetic Information- and Protein-Processing and Amino Acid Metabolism

This group includes 24 genes that showed differential transcription in at least one of the sampling times (Table 1). These genes code for enzymes involved in amino acid metabolism (seven genes), proteins involved in transcription (eight genes, including five sigma factors), the ribosomal protein L28 (*SAVERM2675*), two putative acetyltransferases of ribosomal proteins (*SAVERM703* and *SAVERM758*), and enzymes involved in protein processing (five genes) (Supplementary Materials Table S1).

Interestingly, while sigma factors *sig10* (*SAVERM898*), *sig13* (*SAVERM997*), and *sig60* (*SAVERM213*), and ribosomal proteins acetyltransferases *SAVERM703* and *SAVERM758* showed increased transcription levels in the mutant, *sig32* (*SAVERM3888*), *sig40* (*SAVERM4561*), the L28 ribosomal protein encoding gene *rpmB1*, and the *whiB*-like transcriptional factor *wblE* were clearly underexpressed in the mutant. The Wbl family of transcriptional factors is exclusive of actinobacteria, and their members have been correlated with diverse roles in morphological differentiation and secondary metabolism [11,12].

Notably, the genes *rocA* (*SAVERM2723*) and *putA* (*SAVERM2724*), which encode delta-1-pyrroline-5-carboxylate dehydrogenase and proline dehydrogenase, respectively, and that have been related to proline catabolism [13], and *rocD2* (*SAVERM7112*) and *SAVERM4551*, which encode putative ornithine aminotransferases and are also involved in proline metabolism, were underexpressed in the mutant, while *leuB* (*SAVERM2718*), which is involved in valine, leucine, and isoleucine biosynthesis; *paaI* (*SAVERM1986*), which encodes the phenylacetic acid thioesterase; and putative cysteine desulfurase *SAVERM1061* were overexpressed.

#### 2.1.2. Genes Involved in Nucleotide and Vitamin Metabolism, and DNA Replication, Recombination, and Repair

Eighteen genes falling into this category were found to be differentially transcribed in the mutant (Table 1). Ten of them are involved in DNA replication, recombination, and repair. Of these, seven putative transposases belonging to different families showed an enhanced transcription in the mutant. Additionally, two genes involved in DNA repair, *ku2* (*SAVERM879*), which is probably involved in non-homologous DNA end-joining [14], and *uvrD1* (*SAVERM3463*) that codes for a putative ATP-dependent helicase, were also upregulated. Conversely, *int12* (*SAVERM4626*), which encodes a tyrosine-family recombinase/integrase, showed reduced transcription levels at the stationary phase.

The remaining genes were differentially transcribed only in the exponential phase. Four genes are involved in vitamin metabolism, three of them with lower transcription in the mutant, including cobalamin methylase *cobJ* (*SAVERM6407*), adenosyltransferase *cobA* (*SAVERM6413*), and alkaline phosphatase *phoA* (*SAVERM5915*), which besides being part of the PhoRP two-component system [15] is also involved in folate metabolism. The fourth gene, *thiC* (*SAVERM4265*), is a thiamine biosynthesis protein (Supplementary materials Table S1). The remaining genes are involved in purine metabolism, including *pgmA*, *purA*, and *purN*, all with an enhanced transcription, and *cpdB*, possessing a lower transcription.

#### 2.1.3. Carbohydrate Metabolism Genes

Thirteen genes fall into this category, including four most likely belonging to the same operon (*SAVERM1009*, *galE5*, *mpg2*, and *SAVERM1014*) and putatively involved in galactose metabolism, and showing an enhanced transcription in the mutant. Other genes involved in the metabolism of this sugar were the alpha-galactosidase *agaB1* (*SAVERM1082*), which was underexpressed in the mutant, and the phosphoglucomutase *pgmA* (*SAVERM803*), which showed the opposite behavior. Interestingly, three genes of the tricarboxylic acid/glyoxylate cycle (citrate synthase *citA2*, citrate lyase *citE2*, and methylmalonyl-CoA mutase *meaA1*) were overexpressed in the mutant (Supplementary materials Table S1).

#### 2.1.4. Lipid Metabolism Genes

Nine genes related to lipid metabolism were differentially transcribed. These include the putative 3-oxoacyl-ACP synthase II *fabB2* (*SAVERM2944*), the acyl carrier protein *fabC4* (*SAVERM217*), the enoyl-CoA hydratase *echA1* (*SAVERM492*), and the acetyl/propionyl CoA carboxylase alpha subunit *accA2* (*SAVERM3866*), which are all presumably involved in fatty acid biosynthesis, and the 1-acylglycerol-3-phosphate O-acyltransferase *plsC1* (*SAVERM1485*) putatively involved in glycerophospholipid biosynthesis, among others. Interestingly, all these genes showed increased transcription in the mutant during the exponential phase except *fabB2*, which was underexpressed (Supplementary materials Table S1). However, during the stationary phase, *fabB2* also showed enhanced transcription.

Notably, the direct binding of the PteF orthologue PimM to the promoters of two of these genes has been already demonstrated [7]; thus, they have been included in Supplementary materials Table S1, although they did not meet the statistical criteria. These were the acyltransferase *plsC1* [16] whose transcription was increased in the mutant (Mc 0.88, uncorrected *p*-value 0.0471) and *fabB2* whose transcription was reduced (Mc −0.84, uncorrected *p*-value 0.0410 in t1) or increased (Mc 1.12, *p*-value 0.0048 in t2) depending on the growth phase.

#### 2.1.5. Energy Production Genes

Only three genes belonging to this group were found to be differentially transcribed in the mutant. All of them are involved in oxidative phosphorylation and have a reduced transcription in the mutant: two of them belong to the operon *nuo* (*nuoJ1* and *nuoK1*), and the other one is the ATP synthase *atpF* (Supplementary Materials Table S1). Interestingly, all the genes belonging to the *nuo* operon (*SAVERM4837*-*SAVERM4850*), although in several cases not meeting the statistical criteria, showed the same decreased transcription profile in the mutant.

#### 2.1.6. Transport and External Signals Processing

This group includes 25 genes that showed differential transcription in at least one of the sampling times (Table 1). Interestingly, twelve of them code or participate in the formation of ATP-binding cassette transporters (Supplementary Materials Table S1). Of these, four are putatively involved in sugar transport (*SAVERM1804*, *SAVERM2246*, *SAVERM2247*, and *SAVERM2609*) and showed reduced transcription in the mutant.

Four transporters belonging to the major facilitator superfamily showed differential transcription in the mutant: *SAVERM2455* with a reduced transcription, *SAVERM610*, the sulfate transporter *SAVERM4600*, and *SAVERM6941* with an enhanced transcription.

Notably, in agreement with the enhanced transcription of *SAVERM610*, the genes *fecC1* (*SAVERM600*) and *fecB* (*SAVERM602*), which constitute part of a putative ABC transporter iron(III)/siderophore transport system, were also overexpressed. Based on protein similarity, *SAVERM600-602* could constitute an ABC transport system homologous to the system FecBCD from *E. coli* involved in iron dicitrate transport [17]. The *SAVERM600* and *SAVERM611* genes flank a gene cluster involved in the biosynthesis of the siderophore nrp6 whose expression is also upregulated in the mutant (see below and Table 2 and Supplementary Materials Table S1). Altogether, these results suggest that the ABC system *SAVERM600-602* and the transporter *SAVERM610* would be involved in iron transport using the siderophore nrp6. These transcriptomic results are further supported by the direct binding of PimM to the promoters of *SAVERM602* and *SAVERM610* [7].

#### 2.1.7. Genes Involved in Cell Envelope Biosynthesis and Morphological Differentiation

This group includes eleven genes that showed differential transcription in at least one of the sampling times. These genes code for enzymes involved in cell envelope biosynthesis (the N-acetylmuramoyl-L-alanine amidase *ampD1*), and morphological differentiation (eight genes). The latter are particularly interesting because in *Streptomyces* morphological differentiation is usually accompanied by physiological differentiation [18]. The differential expression of the genes involved in morphological differentiation was somewhat expected given that *S. avermitilis* Δ*pteF* mutants show a delay in spore formation [8].

Our results indicate that the transcriptional regulators *wlbE* and *bldC* that are associated with deficient phenotypes in spore formation (*white*) and in aerial mycelium development (*bald*), respectively, are underexpressed in the mutant. Similarly, the secreted subtilisin inhibitor *sit2* involved in morphological differentiation via *sigU* in *S. coelicolor* [19], and *SAVERM2505* that encodes a DNA-binding protein orthologous to *S. lividans* transcriptional regulator ClgR, which controls the expression of ATP-dependent protease Clp involved in morphological differentiation [20], are also downregulated (Supplementary Materials Table S1). Interestingly, the *clpC1* gene had also been proposed as a direct PteF molecular target given the PimM binding to its coding region [7].

Conversely, the gene *ctpB*, which encodes a cation-transporting P-type ATPase involved in *Bacillus subtilis* sporulation activation [21]; the gene *mreC*, needed for spore cell-wall synthesis in *S. coelicolor* [22]; and both *kipI* and its antagonist *kipA*, which have been involved in sporulation control in *B. subtilis* [23,24], showed enhanced transcription in the mutant (Supplementary Materials Table S1).

#### 2.1.8. Regulatory Genes

As described here, a large set of genes with diverse functions are under the control of PteF, including several regulatory genes listed in the categories described above. This prompted us to analyze other possible transcriptional regulators differentially expressed in the mutant, as these could be mediators of the regulatory control. A complete list of the regulatory genes whose expression is affected in the mutant is presented in Supplementary Materials Table S1.

A total of 31 transcriptional regulators showed a significant differential transcription in the mutant when compared with the parental strain. Such a large number reflects the pleiotropic nature of PAS-LuxR regulators [7,8,25], and probably justifies all the biological processes affected by the mutation (see the functional categories listed above).

Among the regulators controlled by PteF, it is interesting to highlight eight directly involved in diverse secondary metabolites’ biosynthesis control, namely, *avaL2* (*SAVERM2268*) and *avaL1* (*SAVERM2270*), both TetR-family regulators putatively involved in the biosynthesis of a γ-butyrolactone [26]; *avaR1* (*SAVERM3705*), which encodes the avenolide receptor protein [27,28,29]; *olmRII* (*SAVERM2901*) and *olmRI* (*SAVERM2902*), both LuxR-family positive regulators of macrolide oligomycin biosynthesis [30]; *pteR* (*SAVERM410*), the SARP-LAL regulator of the polyene macrolide filipin biosynthesis [8,26,31]; *aveR* (*SAVERM935*), a LAL-family positive regulator of avermectin biosynthesis [32]; and *SAVERM2989*, a MarR-family transcriptional regulator from the neopentalenolactone biosynthetic cluster [26]. All these regulatory genes showed decreased transcription in the mutant, except for *pteR* and *aveR*, which were overexpressed (Table 2 and Supplementary Materials Table S1).

Interestingly, the expression of the *olmRI* and *olmRII* genes had already been proven to be negatively affected by the lack of PteF [7]. Furthermore, *pteF*-deletion mutants showed a severe loss of oligomycin production, whereas the gene complementation of the mutant restored the parental-strain’s phenotype, and gene duplication in the wild-type strain boosted oligomycin production [7]. Similarly, *pteR* has also been reported as a PteF molecular target, via the action of another hierarchical regulator that would be activated by PteF [8].

Besides the abovementioned regulators, other noteworthy findings include the identification of *SAVERM2301*, which codes for a RedD orthologue; the transcriptional activator of the undecylprodigiosin pathway in *S. coelicolor* [33]; *bldC* (*SAVERM4130*), a MerR-family regulator involved in the morphological differentiation and secondary metabolite production in *S. coelicolor* [34]; and *cutS* (*SAVERM2404*), a sensor kinase involved in actinorhodin biosynthesis in *S. lividans* [35], all of them being down-regulated in the mutant (Supplementary Materials Table S1).

#### 2.1.9. Secondary Metabolite Genes

The functional group more clearly affected by *pteF*’s deletion was that of the genes involved in secondary metabolite biosynthesis (Table 1). In this category, when one or more genes critical for metabolite biosynthesis were found statistically significant, the transcription of the other genes belonging to the same cluster with uncorrected *p*-values < 0.05 was also considered significant. Following this broader criterion, sixty-one genes belonging to this group, regardless of the regulatory genes mentioned above, showed a significant differential transcription in the mutant when compared with the parental strain in at least one of the sampling times (Supplementary Materials Table S1). Notably, almost all the genes were detected at the exponential-growth phase. In particular, those related to secondary metabolism precursor biosynthesis were only detected at this sampling time. These genes were: the ornithine aminotransferases *rocD3* (*SAVERM2285*) and *rocD2* (*SAVERM7112*), and the proline dehydrogenase *putA* (*SAVERM2724*), which were underexpressed, and the phosphoglucomutase *pgmA* (*SAVERM803*), the 3-isopropylmalate dehydrogenase *leuB* (*SAVERM2718*), the phosphoribosylglycinamide formyltransferase *purN* (*SAVERM3445*), and the putative citrate synthase *citA2* (*SAVERM3859*), which were overexpressed.

However, the most striking result of the microarray analyses was the identification of differential transcription in 67 genes (including regulatory genes) belonging to 10 out of the 38 putative secondary metabolite gene clusters encoded by *S. avermitilis* genome [26]. Table 2 includes the transcriptional values of the genes belonging to differentially expressed secondary metabolite gene clusters. For the gene cluster boundaries’ definition, we used the StrepDB database [36] in conjunction with information described by Ikeda et al. [26].

The secondary metabolites whose biosynthesis would be affected by *pteF* deletion were of different natures, and included the polyketides filipin (*pte*), oligomycin (*olm*), avermectin (*ave*), and the product of *pks3*; the non-ribosomal peptides nrp3 and the siderophore nrp6; the vibrioferrin-like polyhydroxycarboxylate siderophore *avs*; the terpenoid neopentalenoketolactone (*ptl*); the γ-butyrolactone (*gbl*); and melanin (*melC-1*).

In all these clusters, the differential transcription of at least one key biosynthetic gene was observed. The number of genes affected were: 11 in the *nrp6* cluster (out of 12), 10 (out of 13 and 14 respectively) in the case of the filipin and oligomycin clusters, 8 (out of 11) in the case of the *pk3* cluster, 7 in the case of the avermectin (out of 19) cluster, 6 in the *nrp3* cluster (out of 10), 6 in the *ptl* cluster (out of 14), 5 (out of 5) in the *gbl* cluster, and 2 in the *avs* (out of 4) and melanin *melC-1* (out of 2) clusters (Table 2).

**Figure 3:**
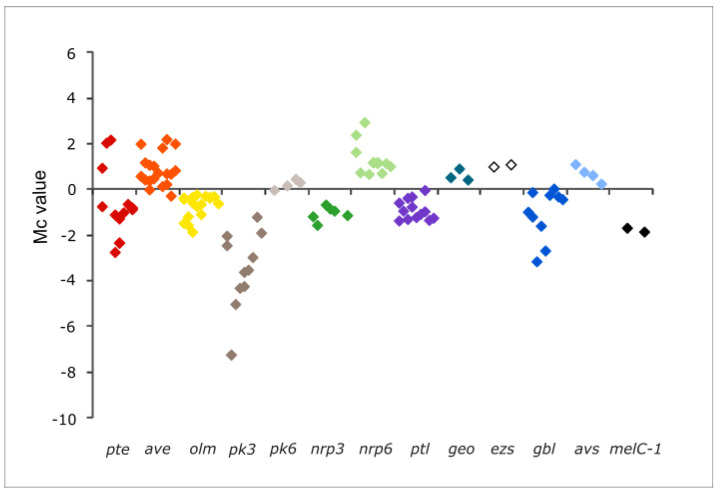
Transcription profiles of secondary metabolite gene clusters genes in *S. avermitilis* Δ*pteF*. Only clusters whose transcription was affected by the mutation are included. All the genes of a given cluster are shown in the plot, including regulatory genes. Colored squares are the plots of differential transcription values for individual genes in the mutant. *pte*, filipin (red); *ave*, avermectin (orange); *olm*, oligomycin (yellow); *pk*, polyketide (gray); *nrp*, non-ribosomal peptide (green); *ptl*, neopentalenoketolactone (purple); *geo*, geosmin (teal); *ezs*, albaflavenol/albaflavenone (white); *gbl*, γ-butyrolactone (dark blue); *avs*, vibrioferrin-like siderophore (light blue); *melC-1*, melanin (black).

Furthermore, a closer look at the transcription of the remaining genes of each of these clusters revealed that most of the genes of a given cluster followed the same tendency. Figure 3 shows the transcription profiles of the secondary metabolite gene clusters genes affected by the mutation including the regulatory genes, and Table 2 provides the transcription values observed for each of the genes.

Seven of the secondary metabolite gene clusters showed an overall reduced transcription, including filipin *pte*, oligomycin *olm*, neopentalenoketolactone *ptl*, and melanin *melC-1* clusters; the silent cluster for γ-butyrolactone *gbl*; and the cryptic gene clusters *pk3* and *nrp3*. Conversely, three gene clusters showed an overall enhanced transcription, including the macrolide avermectin *ave*, the siderophore *avs*, and the cryptic non-ribosomal peptide *nrp6* (Figure 3).

Interestingly, besides the genes mentioned above, all the genes belonging to the clusters coding for the terpenoid albaflavenol/albaflavenone (*ezs*), and the cryptic polyketide *pk4*, also followed the same tendency. In these cases, the transcription values did not meet the statistical criteria, but their uncorrected *p*-values were <0.05 in all instances (Table 2). In the case of the *ezs* genes (*SAVERM3031-3032*), they showed an average of two-fold more transcriptions in the mutant, whereas *pk4* genes (*SAVERM7184-7186*) showed between seven- and nine-fold fewer transcriptions than in the parental strain.

### 2.2. Filipin and Oligomycin Production Are Strongly Reduced in S. avermitilis ΔpteF

Although many of the metabolites whose biosynthesis would be affected by *pteF* deletion are of an unknown structure (cryptic) and the others are not produced under laboratory conditions (silent) [26], the production of two of them could be readily monitored in *S. avermitilis* Δ*pteF*. These were the antifungal pentaene filipin, which is encoded by the *pte* cluster where the regulator is situated, and the ATP-synthase inhibitor oligomycin, which is encoded by the *olm* cluster. In both cases, the production of the secondary metabolite was strongly reduced upon the inactivation of the regulatory gene *pteF* (Figure 1). This agrees with the reduced transcription of most of the biosynthetic genes of both clusters (Figure 3). The exceptions were the discrete thioesterase *pteH*, the cholesterol oxidase *pteG*, and the SARP-LAL regulator *pteR* of the filipin cluster, which were overexpressed. These results corroborate our previous observations by RT-qPCR [7,8].

It is worth noting that two direct targets of PteF in the filipin cluster, *pteA1* and *pteA2* [8], do not fall into significant underexpression values in the mutant strain. This is thought to be derived from the stringent criterion used for defining the statistically significant genes, although we cannot exclude the possibility of an effect on the expression of those genes by any of the 33 transcriptional regulators affected upon the mutation of *pteF*.

### 2.3. Validation of Microarray Results by Using Quantitative RT-PCR

Quantitative RT-PCR was used on the reversed-transcribed RNA samples to confirm that the differential expression indicated by the microarray data was supported by an independent method. The selected genes covered a wide range of expression, including up-regulation and down-regulation. Twelve genes were validated, including genes for the biosynthesis of filipin (*pteC*, *pteB*, *pteR*, and *pteG*), oligomycin (*olmRI*, *olmRII*, and *olmB*), avermectin (*aveR*), the isomerase of the *pk3* cluster (*SAVERM2273*), one ABC transporter of the *nrp6* cluster (*fecB*), the alpha galactosidase *agaB1*, and the heat shock internal membrane protease *htpX1* (*SAVERM4891*).

Overall, the RT-qPCR data and microarray data showed a good concordance (Supplementary Materials Figure S1). The range of dynamics for the relative log_2_ fold change obtained from the RT-qPCRs (−6.53 to +7.54) was higher than that obtained from Mc values from microarrays (−7.24 to +2.94), indicating that RT-qPCRs are more sensitive. This probably reflects on the Pearson’s correlation coefficient (*R*^2^) for the plot, resulting in a lower value than what could be expected. Nevertheless, the obtained value (*R*^2^ = 0.892) still indicates a good correlation of results.

### 2.4. Concluding Remarks

Until now, PAS-LuxR regulator-encoding genes have been found only in polyene macrolide gene clusters, thus constituting a hallmark of these types of clusters. In this context, they are transcriptional activators essential for the biosynthesis of the polyene encoded within the cluster. Their expression is a bottleneck in the biosynthesis of antifungals; thus, polyene production is easily incremented upon a gene dosage increase [25]. Additionally, the heterologous gene complementation of mutants restores the strain’s ability to produce the antifungal compound, thus proving that these regulators are highly conserved [6]. Recently, we have obtained evidence indicating that although these regulators were initially thought to be pathway-specific, they are actually regulatory proteins with a wider range of connotations in addition to polyene biosynthesis. Thus, PteF, the regulator of filipin biosynthesis, was proven to control oligomycin production in *S. avermitilis* [7]. This prompted us to propose that the introduction of PAS-LuxR-regulatory genes into *Streptomyces* species could prove useful for the awakening of dormant secondary metabolite biosynthetic genes [7,8]. This hypothesis was confirmed when PimM, the archetype of the PAS-LuxR regulators, was introduced into *S. albus* J1074, and the production of the hybrid non-ribosomal peptide-polyketide antimycin was activated [9]. Recently, a similar result has been described in *S. albus* S4, where a PimM orthologue (the candicidin regulator FscRI) was identified as necessary for antimycin production [37].

Herein, we have studied the transcriptome of an *S. avermitilis* Δ*pteF* mutant in comparison with that of its parental strain. Our results corroborate our previous observations [7,8], reinforcing the idea that PAS-LuxR regulators control many different cellular processes of bacterial metabolism at the transcriptional level, but particularly stress the importance of PAS-LuxR’s involvement on secondary metabolite biosynthesis.

Notably, 10 (or 12 if we include *ezs* and *pk4* gene clusters) out of the 38 putative secondary metabolite gene clusters encoded by *S. avermitilis* genome [26] showed altered expression in the mutant. In some instances, the modified expression of biosynthetic genes of a given cluster could be explained by the effect of the mutation on the expression of one or more cluster-situated regulators. This is the case of the *aveR* regulator of the avermectin *ave* cluster, the regulators *avaL1* and *avaL2* of the γ-butyrolactone gbl cluster, the oligomycin regulators *olmRI* and *olmRII*, and the MarR regulator (*SAVERM2989*) of the pentalenolactone *ptl* cluster. AveR, the transcriptional activator of avermectin biosynthesis [32], is overexpressed four-fold in the mutant and concomitantly the remaining genes of the *ave* cluster showed enhanced transcription. Conversely, OlmRI and OlmRI, positive regulators of oligomycin biosynthesis [30], showed decreased transcription in the mutant (Mc values −1.56 and −1.47, respectively), and so did the remaining genes of the cluster. It is not known whether AvaL1 and AvaL2 are positive regulators, but it is conceivable given that they show reduced transcription values upon the mutation of the *pteF* gene (fold changes of 6.4 and 8.9, respectively) together with the remaining genes of the *gbl* cluster, including the γ-butyrolactone synthase *avaA*. Both AvaL1 and AvaL2 show convincing similarity to γ-butyrolactone receptor proteins, and although these proteins normally act by repressing the transcription of the synthase gene [38,39,40], there are cases that display the opposite behavior, such as FarA from *S.* lavendulae, which activates the transcription of the synthase *farX* [41]. The same occurs with the MarR regulator of the *ptl* cluster [26] whose transcription is diminished (2-fold) in the mutant as well as that of all *ptl* genes. In the remaining gene clusters, there are no cluster-situated regulatory genes; thus, the effect of the mutation must be explained either by the direct action of PteF on key biosynthetic genes or via the action of other regulatory proteins. In this sense, 30 regulatory genes not situated in the clusters indicated above, most of them with unknown function, were differentially expressed upon the mutation of *pteF* (Supplementary Materials Table S1).

Previous studies have already demonstrated that PAS-LuxR regulators bind a specific conserved sequence [6], which has been found in 97 sites in the genome of *S. avermitilis* outside the filipin cluster [7]. Of these potential binding sites, only 43 were situated in upstream regions of target genes. Among these genes affected by the putative direct binding of PteF, we found that 19 have their expression differentially changed in the microarray data, indicating that PteF effectively controls these processes directly. These include *olmA1* and *olmA2*, and the *fecB* and *SAVERM610* genes from the oligomycin and nrp6 gene clusters, respectively, but also one regulatory gene, namely, the ClgR transcriptional regulator *SAVERM2505*. Interestingly, this regulator has been implicated with morphological and physiological differentiation in *Streptomyces* [20,42] and with proteolysis and DNA repair in *Corynebacterium glutamicum* [43]. Other regulatory genes that show good *p*-values and a high fold-change—although not meeting the strict statistical criteria, and that could also constitute direct targets of PetF—are *SAVERM4561* and *SAVERM6982* (fold-changes of 1.75 and 1.5, respectively) (Supplementary Materials Table S1). These regulators encode an RNA polymerase σ24 factor and a MerR regulator. While the σ-factor targets are unknown, it is expected that the transcription of several genes can be affected. Conversely, the MerR regulator has been shown to regulate *Streptomyces* development [44]. In the absence of novel evidence, the remaining regulatory genes differentially expressed upon mutation are thought to be controlled by *pteF* indirectly given that they do not show binding sequences in their upstream regions.

To our knowledge, this is the second time a genome-wide transcriptomic study has been conducted to describe the pleiotropic nature of a cluster-situated regulator, including that of the regulator of lincomycin biosynthesis LmbU from *S. linconensis* [45]. The cross-regulation of disparate natural-product biosynthetic gene clusters by a cluster-situated regulator has already been described by several groups, although not in genome-wide studies [7,37,46]. Moreover, the ability of some of these regulators to modulate the effects of regulators that act more globally [47], as well as the competition between global regulators [48], have also been reported. Our findings extend further and indicate that PAS-LuxR regulators should be considered wide domain regulators. They affect the expression of multiple genes involved in both primary and secondary metabolism.

The findings reported herein should provide important clues to understanding the intertwined regulatory machinery that modulates the antibiotic biosynthesis in *Streptomyces*, and suggest that the heterologous expression of PAS-LuxR regulators is likely to represent a powerful general strategy for the discovery of novel bioactive natural products.

## 3. Materials and Methods

### 3.1. Strains and Cultivation

*S. avermitilis* NRRL 8165 and its mutant *S. avermitilis* Δ*pteF* [8] were routinely grown and sporulated as described elsewhere [49].

### 3.2. Nucleic Acid Extractions

RNA was extracted as described elsewhere [8]. Briefly, 2 mL from liquid cultures in YEME medium without sucrose was harvested by centrifugation and immediately frozen by immersion in liquid nitrogen. Cells were resuspended in lysis solution [600 µL RLT buffer (RNeasy mini kit; Qiagen); 6 µL 2-mercaptoethanol] and disrupted using a sonicator (Ultrasonic processor XL; Misonix Inc., Farmingdale, New York, NY, USA). RNeasy^®^ Mini kit (Qiagen, Hilden, Germany) was used for RNA isolation using RNase-Free DNase Set (Qiagen, Hilden, Germany) as specified by manufacturer, followed by two consecutive digestions with TURBO^TM^ DNase from Ambion^®^ according to the manufacturer’s instructions. Total RNA concentration was determined with a NanoDrop ND-1000 spectrophotometer (Thermo Scientific, Waltham, MA, USA), and quality and integrity were checked in a Bioanalyzer 2100 apparatus (Agilent Technologies, Santa Clara, CA, USA). Total genomic DNA (gDNA) was isolated from stationary phase cultures following the salting-out procedure [50].

### 3.3. Microarray Hybridizations

The microarray experiment was performed using a common reference design [51]. The microarray chip Custom Gene Expression Microarray, 8 × 15 K (Agilent) was customized to include different sets of probes, as indicated elsewhere [52]. For each microarray hybridization, 10 pmol of Cy3-labelled cDNA obtained from total RNA were mixed with 80 pmol of Cy5-labelled genomic DNA as the common reference. Labelling, hybridization, washing, and scanning conditions were carried out as indicated previously [53]. Three biological replicates from independent cultures were made for each experimental condition. Probe design and gene annotation were performed using the publicly available *S. avermitilis* NRRL 8165 genome sequence with the accession number BA000030.4.

### 3.4. Identification of Differentially Transcribed Genes

Microarray data were normalized and analyzed with the Bioconductor package LIMMA (Linear Models for Microarray Analysis) [54,55]. Spot quality weights were estimated as indicated in the Supplementary section (Tables S2 and S3). Both local and global normalizations were used [56]. Firstly, weighted medians of log2 Cy3/Cy5 intensities were calculated for print-tip correction and afterwards global Loess was applied [57]. The normalized log2 of the Cy3/Cy5 intensities is referred to in this work as the Mg value, which is proportional to the abundance of transcripts for a particular gene [58]. The information from the within-array spot duplicates [55] and empirical array weights [59] were considered in the linear models [54]. The Mg transcription values of the four experimental conditions were compared using two contrasts, mutant versus wild type, corresponding to the two studied growth phases (exponential and stationary). For each gene, the Mc value is the binary log of the differential transcription between the mutant and the wild strain. The Benjamini–Hochberg (BH) false-discovery rate correction was applied to the *p*-values. A positive Mc value indicates upregulation, and a negative one, downregulation. For each contrast, a result was considered statistically significant if the BH-corrected *p*-value was <0.05. However, on certain occasions when the transcription profile of a gene matched that of genes statistically significant and functionally related, or for comparison with previous published results obtained by RT-qPCR or by EMSA assays [7,8], we used an uncorrected *p*-value with a level of significance <0.05.

The microarray data have been deposited in the National Center for Biotechnology Information-Gene Expression Omnibus under accession number GSE185887.

### 3.5. Assessment of Filipin and Oligomycin Production

Filipin production was quantified as described elsewhere [39], whereas oligomycin was measured following the procedure described by Vicente et al. [7].

### 3.6. Reverse Transcription-Quantitative PCR

Reverse transcription of total RNA was performed on selected samples with 5 µg of RNA and 12.5 ng/µL of random hexamer primer (Invitrogen, Waltham, MA, USA) using SuperScript™ III reverse transcriptase (Invitrogen, Waltham, MA, USA) as described previously [60]. Reactions were carried out on two biological replicates with three technical replicates each and appropriate controls were included to verify the absence of gDNA contamination in RNA and primer-dimer formation. Primers (see Supplementary Materials Table S4) were designed to generate PCR products between 97 and 153 bp, near the 5′ end of mRNA. The PCR reactions were initiated by incubating the sample at 95 °C for 10 min followed by 40 cycles at 95 °C for 15 s, 62–70 °C (depending on the set of primers used) for 34 s, and 72 °C for 30 s. To check the specificity of real-time PCR reactions, a DNA melting curve analysis was performed by holding the sample at 60 °C for 60 s followed by slow ramping of the temperature to 95 °C. Baseline and threshold values were determined by the StepOnePlus software. Ct values were normalized with respect to *rrnA1* mRNA (encoding 16S rRNA). Relative changes in gene expression were quantified using the Pfaffl method [61] and the REST© software [62]. The corresponding real-time PCR efficiency (E) of one cycle in the exponential phase was calculated according to the equation E = 10 [−1/slope] [63] using 5-fold dilutions of genomic DNA ranging from 0.013 to 40 ng (*n* = 5 or 6 with three replicates for each dilution) with a coefficient of determination *R*^2^ > 0.99 (Supplementary Materials Figure S2).

## Figures and Tables

**Table 1:** Differential transcription and functional classification of genes affected by *pteF* deletion. The number of genes that are under- (↓) or over-expressed (↑) are indicated.

	Genes Underexpressed ^a^	Genes Overexpressed ^a^	Total
Exponential phase (t1)	63	145	208
Stationary phase (t2)	35	64	99
**Identified Genes ^b^**			
**Function**		**t1**	**t2**
Genetic information- and protein-processing; amino acid metabolism		20 (7 ↓; 13 ↑)	11 (5 ↓; 6 ↑)
Nucleotide and vitamin metabolism; DNA replication, recombination, and repair		16 (4 ↓; 12 ↑)	4 (1 ↓; 3 ↑)
Carbohydrate metabolism		13 (3 ↓; 10 ↑)	1 (1 ↓)
Lipid metabolism		8 (1 ↓; 7 ↑)	4 (4 ↑)
Energy production		2 (2 ↓)	1 (1 ↓)
Transport and external signals’ processing		20 (12 ↓; 8 ↑)	8 (4 ↓; 4 ↑)
Cell envelope biosynthesis and morphological differentiation		9 (4 ↓; 5 ↑)	5 (4 ↓; 1 ↑)
Regulation		27 (12 ↓; 15 ↑)	12 (3 ↓; 9 ↑)
Secondary metabolism		60 (34 ↓; 26 ↑)	6 (2 ↓; 4 ↑)
Miscellaneous		38 (11 ↓; 27 ↑)	19 (1 ↓; 18 ↑)

^a^ Only statistically significant genes with a fold-change value equal higher to ±2 are included. ^b^ All identified genes were accounted for.

**Table 2:** Transcriptional values of genes belonging to differentially expressed secondary metabolite gene clusters in *S. avermitilis ∆pteF* when compared to its parental strain. (t1). The *p*-values are indicated in bold type when found statistically significant. Mc values higher than 1 and their corresponding fold-change above 2 are also in bold.

Gene		Description	Fold-Change	Mc	Corrected *p*-Value	*p*-Value
**Filipin cluster (*pte*)**						
**407**	*pteH*	Thioesterase	1.93	0.95	0.1395	**0.0076**
**408**	*pteG*	cholesterol oxidase	**4.14**	**2.05**	**0.0025**	0.0000
**410**	*pteR*	SARP-family transcriptional regulator	**4.53**	**2.18**	**0.0009**	0.0000
**411**	*pteE*	Ferredoxin	2.14	**−1.10**	0.2533	**0.0233**
**412**	*pteD*	cytochrome P450 monooxygenase	**6.68**	**−2.74**	**0.0005**	0.0000
**413**	*pteC*	cytochrome P450 monooxygenase	**5.03**	**−2.33**	**0.0004**	0.0000
**414**	*pteB*	Dehydrogenase	**5.06**	**−2.34**	**0.0000**	0.0000
**415**	*pteA5*	modular polyketide synthase	2.01	**−1.01**	0.1136	**0.0054**
**416**	*pteA4*	modular polyketide synthase	**2.43**	**−1.28**	**0.0095**	0.0002
**417**	*pteA3*	modular polyketide synthase	1.56	−0.64	0.3514	**0.0457**
**418**	*pteA2*	modular polyketide synthase	1.83	−0.87	0.3639	0.0506
**419**	*pteA1*	modular polyketide synthase	1.74	−0.80	0.4388	0.0752
**Non-ribosomal peptide-6 (*nrp6*)**						
**600**	*fecC1*	ABC transporter iron(III)/siderophore transport system ATP-binding protein	**5.28**	**2.40**	**0.0003**	0.0000
**601**	*fecD1*	ABC transporter iron(III)/siderophore permease	1.68	0.75	0.5625	0.1290
**602**	*fecB*	ABC transporter iron(III)/siderophore-binding protein	**7.73**	**2.95**	**0.0006**	0.0000
**603**	*nrps6*	non-ribosomal peptide synthetase	1.60	0.68	0.3119	**0.0342**
**604**		hypothetical protein	**2.27**	**1.18**	**0.0224**	0.0005
**605**	*fadD2*	acyl-CoA synthetase	**2.30**	**1.20**	**0.0049**	0.0001
**606**		hypothetical protein	1.64	0.71	0.3525	**0.0464**
**607**		taurine catabolism dioxygenase	**2.22**	**1.15**	**0.0017**	0.0000
**608**	*fabC2*	acyl carrier protein	**2.03**	**1.02**	0.1136	**0.0054**
**609**	*fabH4*	3-oxoacyl-ACP synthase III	**2.22**	**1.15**	0.1182	**0.0058**
**610**		MFS transporter protein	**2.28**	**1.19**	**0.0166**	0.0004
**611**		beta-hydroxylase	**3.12**	**1.64**	**0.0005**	0.0000
**Avermectin cluster (*ave*)**						
**935**	*aveR*	LuxR-family transcriptional regulator	**4.00**	**2.00**	**0.0049**	0.0001
**936**	*aveF*	C-5 ketoreductase	1.51	0.59	0.6016	0.1518
**937**	*aveD*	C5-O-methyltransferase	1.35	0.43	0.6148	0.1603
**938**	*aveA1*	type I polyketide synthase	**2.27**	**1.18**	0.1820	**0.0122**
**939**	*aveA2*	type I polyketide synthase	1.31	0.39	0.7264	0.2537
**940**	*aveC*	post-polyketide modification protein	1.00	0.00	0.9993	0.9950
**941**	*aveE*	cytochrome P450 monooxygenase	**2.06**	**1.04**	0.1376	**0.0074**
**942**	*aveA3*	type I polyketide synthase	1.40	0.49	0.6328	0.1725
**943**	*aveA4*	type I polyketide synthase	1.66	0.73	0.4435	0.0764
**944 ^a^**	*orf-1*	Reductase	1.10	0.14	0.9330	0.6990
**945**	*aveBI*	dTDP-L-oleandrose transferase (glycosyltransferase)	1.62	0.70	0.6052	0.1543
**946**	*aveBII*	dTDP-glucose 4.6-dehydratase	1.17	0.23	0.8027	0.3477
**947**	*aveBIII*	glucose-1-phosphate thymidyltransferase	**2.11**	**1.08**	0.2027	**0.0154**
**948**	*aveBIV*	dTDP-4-keto-6-deoxy-L-hexose 4-reductase	1.21	−0.28	0.8828	0.5032
**949**	*aveBV*	dTDP-4-keto-6-deoxyhexose 3.5-epimerase	1.60	0.68	0.5387	0.1160
**950**	*aveBVI*	dTDP-4-keto-6-deoxy-L-hexose2.3-dehydratase	1.79	0.84	0.4308	0.0711
**951**	*aveBVII*	dTDP-6-deoxy-L-hexose 3-O-methyltransferase	**4.03**	**2.01**	**0.0020**	0.0000
**952**	*aveBVIII*	dTDP-4-keto-6-deoxy-L-hexose 2.3-reductase	**3.56**	**1.83**	**0.0049**	0.0001
**953**	*aveG*	Thioesterase	**4.59**	**2.20**	**0.0018**	0.0000
**Melanin cluster (*melC-1*) ^b^**						
**1136**	*melC1*	tyrosinase co-factor protein	**3.20**	**−1.68**	0.0776	**0.0015**
**1137**	*melC2*	Tyrosinase	**3.61**	**−1.85**	**0.0078**	0.0000
**γ-butyrolactone cluster (*gbl*)**						
**2266**	*avaC*	Phosphatase	1.97	−0.98	0.0794	**0.0030**
**2267**	*avaB*	Oxidoreductase	**2.30**	**−1.20**	0.2777	**0.0279**
**2268**	*avaL2*	TetR-family transcriptional regulator	**8.88**	**−3.15**	**0.0078**	0.0001
**2269**	*avaA*	gamma-butyrolactone biosynthesis protein	**3.01**	**−1.59**	0.1456	**0.0083**
**2270**	*avaL1*	TetR-family transcriptional regulator	**6.41**	**−2.68**	**0.0035**	0.0000
**Polyketide-3 cluster (*pk3*)**						
**2272**		hypothetical protein	**5.46**	**−2.45**	0.2000	**0.0150**
**2273**		Isomerase	**151.17**	**−7.24**	**0.0208**	0.0005
**2274**		secreted protein	**32.45**	**−5.02**	**0.0473**	0.0014
**2275**		transmembrane efflux protein	**19.97**	**−4.32**	0.1124	**0.0053**
**2276**		3-oxoacyl-ACP synthase III	**18.90**	**−4.24**	0.1242	**0.0063**
**2277**		Thioesterase	**12.30**	**−3.62**	0.2222	**0.0183**
**2278**		F420-dependent dehydrogenase	**11.47**	**−3.52**	0.3037	**0.0327**
**2279**		acyl-CoA synthetase	**7.84**	**−2.97**	0.3522	**0.0462**
**2280**	*pks3-1*	modular polyketide synthase	**2.30**	**−1.20**	0.6288	0.1702
**2281**	*pks3-2*	modular polyketide synthase	**3.73**	**−1.90**	0.5893	0.1436
**2282**	*pks3-3*	acyl carrier protein	**4.08**	**−2.03**	0.3974	0.0593
**Oligomycin cluster (*olm*)**						
**2890**	*ccrA1*	crotonyl-CoA reductase	1.34	−0.42	0.4368	0.0743
**2891**		hypothetical protein	**2.27**	**−1.18**	0.2280	**0.0193**
**2892**	*olmA4*	modular polyketide synthase	1.26	−0.33	0.4760	0.0913
**2893**	*olmA5*	modular polyketide synthase	1.16	−0.22	0.6421	0.1789
**2894**	*olmB*	cytochrome P450 monooxygenase	**2.13**	**−1.09**	0.1512	**0.0087**
**2895**	*olmA7*	modular polyketide synthase	1.56	−0.64	0.0746	**0.0028**
**2896**	*olmA6*	modular polyketide synthase	1.67	−0.74	0.1443	**0.0080**
**2897**	*olmA3*	modular polyketide synthase	1.25	−0.32	0.3610	**0.0493**
**2898**	*olmA2*	modular polyketide synthase	1.24	−0.31	0.5785	0.1377
**2899**	*olmA1*	modular polyketide synthase	1.53	−0.61	0.1817	**0.0121**
**2900**		P450-like protein	1.48	−0.57	0.2474	**0.0224**
**2901**	*olmRII*	LuxR-family transcriptional regulator	**2.77**	**−1.47**	0.0712	**0.0026**
**2902**	*olmRI*	LuxR-family transcriptional regulator	**2.95**	**−1.56**	**0.0006**	0.0000
**2903**	*olmC*	Thioesterase	**3.63**	**−1.86**	0.1235	**0.0062**
**Neopentalenolactone cluster (*ptl*)**						
**2989**		MarR-family transcriptional regulator	**2.08**	**−1.06**	**0.0487**	0.0015
**2990**	*gap1*	glyceraldehyde-3-phosphate dehydrogenase	**2.57**	**−1.36**	0.2469	**0.0222**
**2991**	*ptlH*	1-deoxypentalenic acid 11-beta hydroxylase	1.91	−0.93	0.1529	**0.0089**
**2992**	*ptlG*	transmembrane efflux protein	1.29	−0.37	0.9068	0.5988
**2993**	*ptlF*	1-deoxy-11beta-hydroxypentalenic acid dehydrogenase	1.69	−0.76	0.2639	**0.0251**
**2994**	*ptlE*	Baeyer-Villiger monooxygenase	**2.46**	**−1.30**	**0.0087**	0.0001
**2995**	*ptlD*	Dioxygenase	**2.31**	**−1.21**	0.0962	**0.0041**
**2996**	*ptlC*	hypothetical protein	**2.10**	**−1.07**	0.5367	0.1153
**2997**	*ptlB*	farnesyl diphosphate synthase	1.95	−0.96	0.5690	0.1322
**2998**	*ptlA*	pentalenene synthase	**2.53**	**−1.34**	0.5592	0.1269
**2999**	*ptlI*	pentalenene C13 hydroxylase; cytochrome P450	**2.36**	**−1.24**	0.4943	0.0993
**3000**	*ptlR*	AraC-family transcriptional regulator	1.49	−0.58	0.5379	0.1158
**3001**	*ptlJ*	Lyase	1.24	−0.31	0.6109	0.1579
**3002**	*ptlL*	hypothetical protein	1.02	−0.03	0.9754	0.8867
**Albaflavenol/albaflavenone cluster (*ezs*)**						
**3031**	*cyp14*	epi-isozizaene hydroxylase (cytochrome P450 monooxygenase)	**2.00**	**1.00**	0.2918	**0.0301**
**3032**	*ezs*	epi-isozizaene synthase (sesquiterpene cyclase)	**2.14**	**1.10**	0.1621	**0.0097**
**Non-ribosomal peptide-3 cluster (*nrp3*)**						
**3155**		MbtH-like protein	**2.27**	**−1.18**	0.2696	**0.0261**
**3156**	*nrps3-1*	non-ribosomal peptide synthetase	**2.95**	**−1.56**	0.0867	**0.0035**
**3157**		export protein	1.58	−0.66	0.6407	0.1773
**3158**	*nrps3-2*	non-ribosomal peptide synthetase	1.79	−0.84	0.6149	0.1623
**3159**	*nrps3-3*	non-ribosomal peptide synthetase	1.91	−0.93	0.5290	0.1123
**3160**		Aminotransferase	**2.19**	**−1.13**	0.4384	0.0749
**3161**	*dapF2*	diaminopimelate epimerase	**2.04**	**−1.03**	0.5045	**0.1029**
**3162**		hypothetical protein	**2.87**	**−1.52**	0.1668	**0.0101**
**3163**		hypothetical protein	**2.36**	**−1.24**	0.3227	**0.0384**
**3164**		hypothetical protein	**3.01**	**−1.59**	**0.0003**	0.0000
**Polyketide-4 cluster (*pk4*)**						
**7184**	*pks4*	modular polyketide synthase	**9.32**	**−3.22**	0.2980	**0.0315**
**7185**		UDP-glucose:sterol glucosyltransferase	**8.28**	**−3.05**	0.2753	**0.0271**
**7186**	*cyp26*	cytochrome P450 hydroxylase	**7.36**	**−2.88**	0.2419	**0.0213**
**Polyhydroxycarboxylate siderophore cluster (*avs*)**						
**7320**	*avsA*	siderophore synthetase component	**2.16**	**1.11**	**0.0295**	0.0007
**7321**	*avsB*	siderophore synthetase component	1.71	0.77	0.2474	**0.0223**
**7322**	*avsC*	siderophore synthetase component	1.55	0.63	0.4654	0.0862
**7323**	*avsD*	diaminopimelate decarboxylase	1.19	0.25	0.8125	0.3606

^a^ Not involved in avermectin biosynthesis; ^b^ Values from stationary phase (t2) analysis.

## Data Availability

The microarray data are deposited in the National Center for Biotechnology Information-Gene Expression Omnibus under accession number GSE185887.

## Supplementary Materials

The following supporting information can be downloaded at: https://www.mdpi.com/article/10.3390/antibiotics11080994/s1, Figure S1: Validation of microarray results using RT-qPCR; Figure S2: Primer efficiency; Table S1: Differentially expressed genes in *S. avermitilis* Δ*pteF* when compared to its parental strain; Table S2: Determination of the quality flag for array spots; Table S3: Assigned weights to each spot flags; Table S4: Sequence of primers used for qPCR.
